# Comparative studies of genomic and epigenetic factors influencing transcriptional variation in two insect species

**DOI:** 10.1093/g3journal/jkac230

**Published:** 2022-09-07

**Authors:** Xin Wu, Neharika Bhatia, Christina M Grozinger, Soojin V Yi

**Affiliations:** School of Biological Sciences, Institute for Bioengineering and Bioscience, Georgia Institute of Technology, Atlanta, GA 30332, USA; Department of Entomology, Center for Pollinator Research, Huck Institutes of the Life Sciences, Pennsylvania State University, University Park, PA 16801, USA; School of Biological Sciences, Institute for Bioengineering and Bioscience, Georgia Institute of Technology, Atlanta, GA 30332, USA; Department of Ecology, Evolution and Marine Biology, University of California Santa Barbara, Santa Barbara, CA 93106, USA

**Keywords:** gene expression variability, DNA methylation, insects, core promoter elements

## Abstract

Different genes show different levels of expression variability. For example, highly expressed genes tend to exhibit less expression variability. Genes whose promoters have TATA box and initiator motifs tend to have increased expression variability. On the other hand, DNA methylation of transcriptional units, or gene body DNA methylation, is associated with reduced gene expression variability in many species. Interestingly, some insect lineages, most notably Diptera including the canonical model insect *Drosophila melanogaster*, have lost DNA methylation. Therefore, it is of interest to determine whether genomic features similarly influence gene expression variability in lineages with and without DNA methylation. We analyzed recently generated large-scale data sets in *D. melanogaster* and honey bee (*Apis mellifera*) to investigate these questions. Our analysis shows that increased gene expression levels are consistently associated with reduced expression variability in both species, while the presence of TATA box is consistently associated with increased gene expression variability. In contrast, initiator motifs and gene lengths have weak effects limited to some data sets. Importantly, we show that a sequence characteristics indicative of gene body DNA methylation is strongly and negatively associate with gene expression variability in honey bees, while it shows no such association in *D. melanogaster.* These results suggest the evolutionary loss of DNA methylation in some insect lineages has reshaped the molecular mechanisms concerning the regulation of gene expression variability.

## Introduction

Expression levels of the same genes show variability at different levels of biological units, from cells to populations. The widely observed variability of gene expression at different levels is thought to share some common underlying molecular mechanisms (Ho *et al.* 2008; Tirosh *et al.* 2009; Lehner 2010; Li *et al.* 2010; Alemu *et al.* 2014). This variation, which we will refer to as gene expression variability, is implicated in several phenotypic traits such as aging, development, disease, and immunity (Ho *et al.* 2008; Li *et al.* 2010; Hagai *et al.* 2018; Bashkeel *et al.* 2019).

Previous studies of gene expression variability have discovered that highly expressed genes tend to have reduced variability between individuals in diverse taxa (Bird 1995; Choi and Kim 2008; Huh *et al.* 2013; Wu, Lindsey *et al.* 2020). It is hypothesized that natural selection has reduced the expression variability of highly expressed genes due to the advantages associated with the improved control of the inherent stochasticity of transcription and subsequent protein synthesis (Fraser *et al.* 2004; Newman *et al.* 2006; Wang and Zhang 2011; Barroso *et al.* 2018). Genes that are constitutively highly expressed are typically essential housekeeping genes, where noise is therefore minimized by natural selection (Fraser *et al.* 2004; Wang and Zhang 2011; Barroso *et al.* 2018).

Other traits that significantly associated with gene expression variability include genomic features such as gene length, presence of a TATA box, and initiator motifs (Park *et al.* 2012; Huh *et al.* 2013; Ravarani *et al.* 2016; Faure *et al.* 2017; Wu, Lindsey *et al.* 2020). The presence of a TATA box is strongly associated with high gene expression variability, with other core promoter elements such as initiator motifs and GC motifs being associated with higher gene expression variability to a much lesser degree (Faure *et al.* 2017). Therefore, genomic features can play significant roles in shaping gene expression variability.

Gene body DNA methylation, which is an ancestral form of epigenetic regulation in animal genomes, is negatively associated with gene expression variability in humans (Huh *et al.* 2013). Studies in insects, in particular from hymenopteran species including fire ants and wasps, also reported similar observations (Zeng and Yi 2010; Hunt *et al.* 2013; Wang *et al.* 2016; Wu, Lindsey *et al.* 2020). However, the relative contribution of gene body DNA methylation compared to the aforementioned genomic features has not been examined in insects. One of the reasons for this lack of knowledge is due to the fact that the most extensively studied model insect *Drosophila melanogaster*, and other species in the Diptera, lacks DNA methylation which is best explained by lineage-specific loss (Sarda *et al.* 2012; Yi 2012). To address the gap of knowledge, here we examined relative impacts of different genomic features on gene expression variability through a comparative analysis of honey bees (*Apis mellifera*), a hymenopteran lineage possessing ancestral gene body methylation, and *D. melanogaster*. We integrated data from 15 *D. melanogaster* studies and 8 *A. mellifera* studies to comprehensively address these questions (Table 1). Our study confirms impacts of several genomic features on gene expression variability in both species. Intriguingly, our analyses indicate pervasive and significant effects of DNA methylation on gene expression variability in *A. mellifera*. These results provide new insights into regulation of gene expression variability in insects.

**Table 1.** Source datasets for gene expression variability.

Source publication	Sample size	Sample type	GEO accession
*Drosophila melanogaster*			
Genç et al. (2020)	23	Head	GSE153225
Seong et al. (2020)	42	Head, fat body, testes	GSE140950
Wang et al. (2020)	24	Head	GSE109489
Shah et al. (2021)	24	Brain	GSE140663
Lindsey et al. (2021)	48	Whole body	GSE162666
Hood et al. (2020)	24	Whole body	GSE158189
Agren et al. (2020)	72	Whole body	GSE155395
Miozzo and Nagoshi, unpublished results	18	Brain	GSE156890
Thackray et al. (2020)	36	Brain	GSE144028
Brown et al. (2020)	30	Head	GSE144433
Weigelt et al. (2020)	78	Brain, Thorax, Fat Body	GSE130158
*Apis mellifera*			
Shpigler et al. (2017)	180	Mushroom body	GSE85876
Harris et al. (2019)	29	Head and pupa	GSE116629
Liberti et al. (2019)	18	Brain	GSE127185
Rutter and Cook (2020)	24	Whole body	GSE121885
Naeger and Robinson (2016)	48	Mushroom body	GSE85433
Jasper et al. (2020)	38	Fat body and brain	GSE145395
Traniello et al. (2020)	96	Brain	GSE130700
Doublet et al. (2016)	11	Brain	GSE81664

## Materials and methods

### Gene expression data

We analyzed a total of 20 published RNA-seq studies for this study, 12 of which are from fly (*D. melanogaster*) and 8 from honey bee (*A. mellifera*; Table 1). Our *D. melanogaster* datasets were chosen from a diverse set of laboratories as well as recently published with at least 10 samples (no more than 2 years old). The *A. mellifera* studies were all of the RNA-seq datasets we could access, as well as being fairly recent and a minimum of 10 samples (one from 2012, the rest were from 2016 to 2020).

### Data processing

Reads for each study were trimmed to remove low-quality reads and adaptors using default Trim_galore! (Martin 2011) settings. Trimmed reads were then aligned to their respective genomes, amel 4.5 and dmel r6.33 for *A. mellifera* and *D. melanogaster*, respectively, using HISAT2 with soft clipping disabled (parameter setting: –sp 1,000, 1,000). Following alignment, gene counts were generated with HTSeq (Anders *et al.* 2015) default parameters and imported into R (R Core Team 2014) for further downstream analyses. Gene expression for each study was quantified and normalized using the “estimateSizeFactors” function in the DESeq2 package (Love *et al.* 2014). To remove lowly expressed genes, we removed genes with counts less than 5 and also required a gene to be expressed in at least 10% of all samples in the study. Gene expression variation was measured as the % coefficient of variation (CV) of gene expression (Huh *et al.* 2013) and CpG O/E values for the *A. mellifera* genome were calculated as previously described (Lindsey *et al.* 2018).

### Data processing

Core promoter element designations for TATA boxes and initiator motifs were obtained from the Eukaryotic Promoter Database (Cavin Perier *et al.* 1998; Dreos *et al.* 2017). Briefly, promoter classifications for each organism were downloaded from the database using the “EPDnew selection tool” as performed in a previous study (Faure *et al.* 2017).

### Statistics

For our full linear model, gene expression variation was used as the response variable for the following quadratic model: log_10_(CV) ∼ log_2_(expression) + log_2_(expression)^2^ + log_10_(gene length) + TATA box + Initiator motif + X, where X are additional covariates from each experiment based on its metadata file. In our second set of linear models, we first regressed out the effect of gene expression with log_10_(CV) ∼ log_2_(expression) + log_2_(expression)^2^ and then using the residuals as the response variable mirroring the full linear model: residuals ∼ log_10_(gene length) + TATA box + Initiator motif + X. Partial correlation was performed using the “pcorr” function in R with gene expression as the variable that was controlled for and gene length and CpG O/E (*A. mellifera* studies only) as the response variables.

## Results

### Core promoter elements are significant contributors to gene expression variation

For all the data sets (Table 1), we first quantified gene expression variability as the CV of gene expression, as in previous studies (Huh *et al.* 2013; Islam *et al.* 2014; Fan *et al.* 2016). We then evaluated the contributing factors using a linear model using the following covariates: mean gene expression, gene length, presence of a TATA box, and presence of an initiator motif (see *Methods*). Our main motivation was to examine the impact of DNA methylation on gene expression variability. However, for data sets in *A. mellifera*, matching data on DNA methylation and gene expression are sparse, and the existing data sets tend to have small number of biological replicates. Therefore, for *A. mellifera* data sets, we included CpG O/E as an additional covariate which is an approximate measure of DNA methylation (Elango *et al.* 2009). Notably, CpG O/E strongly negatively correlates with the measured DNA methylation levels in honey bee and other invertebrates in literature (e.g. Sarda *et al.* 2012), and also in a compiled data set from *A. mellifera* (Supplementary Fig. 2).

**Fig. 1.**
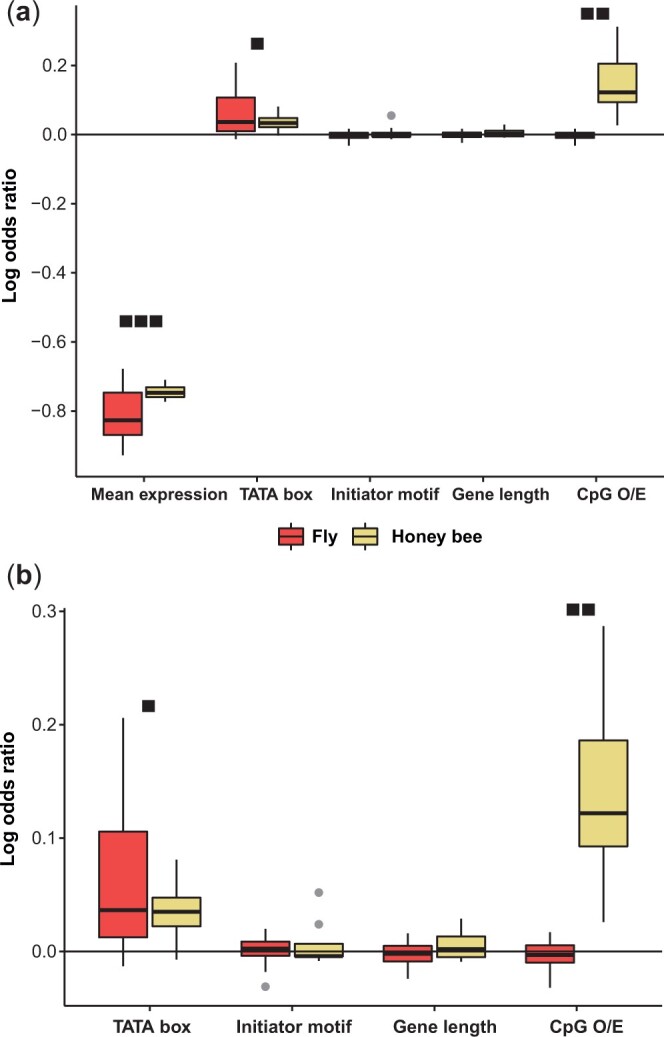
Linear model covariate coefficients summary. We used the coefficient of variation of gene expression as a proxy for gene expression variability and modeled it using a set of key factors hypothesized to affect transcriptional noise. The log odds ratio was calculated for each covariate coefficient (*Methods*) in each study. a) Box plot of log ratio of covariate coefficients for the full linear model including gene expression, presence of a TATA box, presence of an initiator motif, gene length, and CpG O/E. b) Log ratio of covariate coefficients with mean regressed out using a quadratic model (*Methods*). The number of boxes above the boxplots represents the percentage of studies the covariate is significant in. Three boxes mean the covariate is statistically significant (*P* < 0.05) in all studies (100%), 2 boxes represent significance in 50–70% of all studies, and 1 box represents significance in at most 30% of the studies. Boxes for CpG O/E only apply for the *A. mellifera* datasets and not the *D. melanogaster* data sets.

As expected, average gene expression was strongly negatively correlated with gene expression variability and was by far the most significant term with the largest coefficient in the linear model in all datasets (Huh *et al.* 2013; Islam *et al.* 2014; Fan *et al.* 2016; Wu, Lindsey *et al.* 2020) (Fig. 1a and Supplementary Table 1). Following mean expression, the presence of a TATA box in the gene promoter region was a significant term in 12 of the 15 *D. melanogaster* datasets (Supplementary Table 1) and in 6 of the 8 *A. mellifera* datasets (Supplementary Table 1). The presence of a TATA box was positively and significantly correlated with gene expression variability in all but one study (Hood *et al.* 2020). The other core promoter element, presence of initiator motif, was only significant in approximately half of the studies (6 out of 12 *D. melanogaster* studies; 4 out of 8 *A. mellifera* studies; Supplementary Table 1). The direction of correlation for the initiator motif was also less consistent than the previous 2 discussed factors, as the coefficient was positive in 3 of the 6 *D. melanogaster* datasets it was significant in and in 2 of the 4 *A. mellifera* datasets it was significant in (Supplementary Table 1). Lastly, gene length, while a significant term in the majority of datasets, also failed to display a consistent direction of correlation in either *D. melanogaster* or *A. mellifera* datasets. In conclusion, in the linear models, we observed a strong and significant negative correlation between mean expression and expression variability along with consistent, though not always significant, correlation between the presence of a TATA box and expression variability (Fig. 1a). The other promoter element, initiator motifs, and gene length did not show a consistent relationship with expression variability.

Interestingly, CpG O/E, which we used as a proxy for the level of gene body methylation, was highly significantly and positively correlated with gene expression variability in *A. mellifera* data sets (Fig. 1 and Supplementary Tables 1 and 2). Thus, in addition to gene expression, CpG O/E displayed strong and stable correlation with gene expression variability across all *A. mellifera* datasets. Since CpG O/E itself is negatively correlated with DNA methylation, these results imply that gene body DNA methylation is associated with reduced gene expression variability.

Because of the strong effects of mean gene expression on gene expression variability, we applied another strategy to control for this effect. We first regressed out mean expression using a quadratic model (see *Methods*). We used the quadratic model as it was shown to have fairly unbiased residual distributions and previously applied to model the relationship between gene expression and expression variability (Alemu *et al.* 2014). The residual from this regression would reflect the remaining variability independent of the mean gene expression levels, which then can be interrogated for other genomic factors. This analysis yielded almost identical results as our initial linear models (Supplementary Table 2). For the TATA box term, the significance at the *P* < 0.05 threshold and the direction of correlation remained the same for all *A. mellifera* studies. Similarly, the *P*-value for the TATA box term was nearly the same for the *D. melanogaster* datasets, with only one study (Thackray *et al.* 2020), having a small change going from *P* = 0.055 in the full model to *P* = 0.048 (Supplementary Table 2). For the initiator motif term, the direction changed for one study (Thackray *et al.* 2020) while significance remained the same for all *D. melanogaster* studies (Supplementary Table 2). Gene length, as with the other covariates, was the same across all studies with the exception of one study (Brown *et al.* 2020), which was no longer statistically significant after regressing out the effects of gene expression (Supplementary Table 2). Due to the expected strong effects of mean expression on expression variability, there was a sharp drop off in *R*^2^ values across these analyses. Specifically, after regressing out gene expression, only 3 *D. melanogaster* and 2 *A. mellifera* studies had models explaining more than 10% of the variance in expression variability. Nevertheless, the results of both linear model approaches indicate that the presence of a TATA box in the gene promoter region is consistently correlated with higher expression variability (Fig. 1b).

When mean expression is regressed out, the CpG O/E term was the most impactful term in the *A. mellifera* datasets (Fig. 1b and Supplementary Table 2). It was highly significant in all 8 studies (*P* < 0.001 in all cases) with an average coefficient of 0.14 (Fig. 1b and Supplementary Table 2). None of the *D. melanogaster* datasets contained significant CpG O/E terms which was an expected result given the lack of genomic DNA methylation (Fig. 1b; Sarda *et al.*, 2012; Yi, 2012). Therefore, controlling for gene expression yielded similar results suggesting that outside of gene expression itself, DNA methylation had the biggest impact on gene expression variability followed by the core promoter elements (Fig. 1b and Supplementary Table 2).

We also used a partial correlations approach (Kim and Yi 2006; 2007) to examine effects of covariates free from the effects of gene expression. Specifically, we separately applied partial correlations for each numerical variable (gene length for both organisms in addition to CpG O/E for *A. mellifera*) while controlling for mean expression. Using this method, gene length was a significant term in 10 *D. melanogaster* and 6 *A. mellifera* datasets (Supplementary Table 3). CpG O/E was once again highly significant in all 8 *A. mellifera* datasets with an average partial Pearson correlation of 0.157 (Supplementary Table 3).

These results remained (Supplementary Table 4) when we used only a subset of gene sets that are likely to be methylated, using the CpG O/E cutoff of 0.8, following Sarda *et al*. (2012). Furthermore, to test of the results were affected by the tissue heterogeneity, we used a subset of data sets from brain (3 from the fly studies, 3 from honey bee studies, Table 1). Analyses of this subset of data recapitulate the same patterns as seen in the whole data set (Supplementary Fig. 2). Even though we need to further consider additional tissues, this analysis indicates that the significance of gene body DNA methylation on gene expression variability is apparent in the brain data sets from the 2 species. We also examined experimentally determined DNA methylation data sets (Wu, Galbraith *et al.* 2020) and gene expression (Galbraith *et al.* 2015). These data sets are from bees in the same genetic background although the exact individuals are not matched. Using DNA methylation levels instead of CpG O/E, we performed the same statistical analyses. We found that in the full model, DNA methylation level is a highly significant predictor of gene expression variability, followed by the mean expression level (*P *<* *10^−15^ for both terms, Supplementary Table 5). When the expression level is regressed out, DNA methylation was the best predictor of gene expression variability (Supplementary Table 5).

## Discussion

By utilizing the emerging richness of large-scale gene expression data sets, we initiated a study of the genomic and epigenetic factors underlying gene expression variability in a canonical model insect fruit fly (*D. melanogaster*) and the honey bee (*A. mellifera*). Since *D. melanogaster* does not exhibit widespread DNA methylation and *A. mellifera* does, this comparison is useful to ask whether DNA methylation is associated with gene expression variability in insects. While a negative association between DNA methylation and gene expression variability was previously found in 3 studies (Hunt *et al.* 2013; Wang *et al.* 2016; Wu, Lindsey *et al.* 2020), these were limited in sample size and did not control for other genomic features.

We show that, as in other taxa, highly expressed genes tend to have reduced expression variability in both flies and honey bees. In addition, the presence of TATA boxes was often associated with increased noise, consistent with previous studies (Blake *et al.* 2003; Lehner 2008; Ravarani *et al.* 2016; Faure *et al.* 2017). Initiator motifs and gene lengths had some effects in some data sets, but their effects were not consistent across the data sets (Fig. 1).

While the effect of gene expression levels and TATA box and initiator motifs were consistent between *D. melanogaster* and *A. mellifera*, these 2 species differ by the presence (*A. mellifera*) and absence (*D. melanogaster*) of DNA methylation. To contrast the effect of DNA methylation on these 2 species, we utilized CpG O/E as a proxy measurement for gene body DNA methylation (Elango *et al.* 2009). As expected from the lack of DNA methylation in *D. melanogaster* genome, CpG O/E in *D. melanogaster* is unimodally distributed and shows no relationship with gene expression (Fig. 1b). In contrast, in *A. mellifera*, for all of our statistical methods (full linear model, linear model with mean expression regressed out, and partial correlations), the CpG O/E term was highly significantly and positively correlated with gene expression variability (Fig. 1 and Supplementary Tables 1–3). Thus, both mean expression, which was by far the most significant and impactful covariate, and CpG O/E displayed strong and stable correlation with gene expression variation across all *A. mellifera* datasets. Since CpG O/E itself is negatively correlated with DNA methylation (Sarda *et al*. 2012 and Supplementary Fig. 1), these results align with previous findings in both mammals and insects that DNA methylation is associated with reduced gene expression variation (Huh *et al.* 2013; Wu, Lindsey *et al.* 2020). These results strongly imply that gene body DNA methylation is associated with gene expression variability in *A. mellifera*, and its effect is stronger and more consistent than other genomic features such as TATA box, initiator motif and gene lengths.

The molecular mechanism underlying this association between DNA methylation and gene expression variation is not resolved in *A. mellifera*, but studies in other taxa have revealed several potential pathways (Choi and Kim 2009; Coleman-Derr and Zilberman 2012; Huh *et al.* 2013). For example, gene body methylation may directly or indirectly reduce spurious intragenic transcription, by avoiding erroneous intron retention (Horvath *et al.* 2019) or interacting with other epigenetic modifications (Coleman-Derr and Zilberman 2012). Our results beg the question of how *D. melanogaster* regulate expression variability in the absence of DNA methylation. There is no indication of *D. melanogaster* having greater variability of gene expression in our data, and there is very little difference in terms of *R*^2^ of any of the statistical models. It is likely that alternative mechanisms have evolved in *D. melanogaster* to compensate the lack of DNA methylation. For example, gene body DNA methylation in *A. mellifera* and several histone markers in *D. melanogaster* are highly associated (Hunt *et al.* 2013). Comparing taxa where the loss of DNA methylation has occurred recently could provide some insight into the evolution of regulatory mechanisms underlying gene expression variability.

## Supplementary Material

jkac230_Supplementary_Figures

jkac230_Supplementary_Tables

## Data Availability

All data used in this study are found in public domain and the accession numbers are indicated in Table 1. Supplemental material is available at G3 online.
