# Supplementary material for: Comparative studies of genomic and epigenetic factors influencing transcriptional variation in two insect species
Source: G3 (Bethesda). 2022 Sep 7;12(11):jkac230. doi: 10.1093/g3journal/jkac230 (PMC9635643; doi:10.1093/g3journal/jkac230)
Supplement: jkac230_Supplementary_Figures [file jkac230_supplementary_figures.pdf]

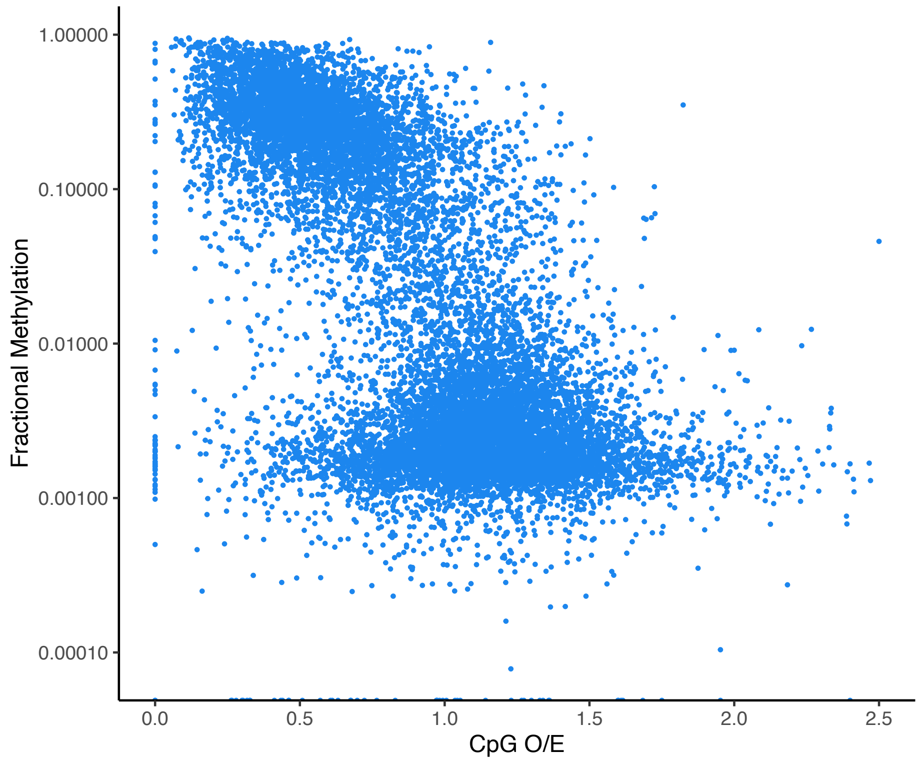

Supplementary Figure 1. CpG O/E and DNA methylation levels are highly negatively correlated in a WGBS data set (Wu et al. 2020).

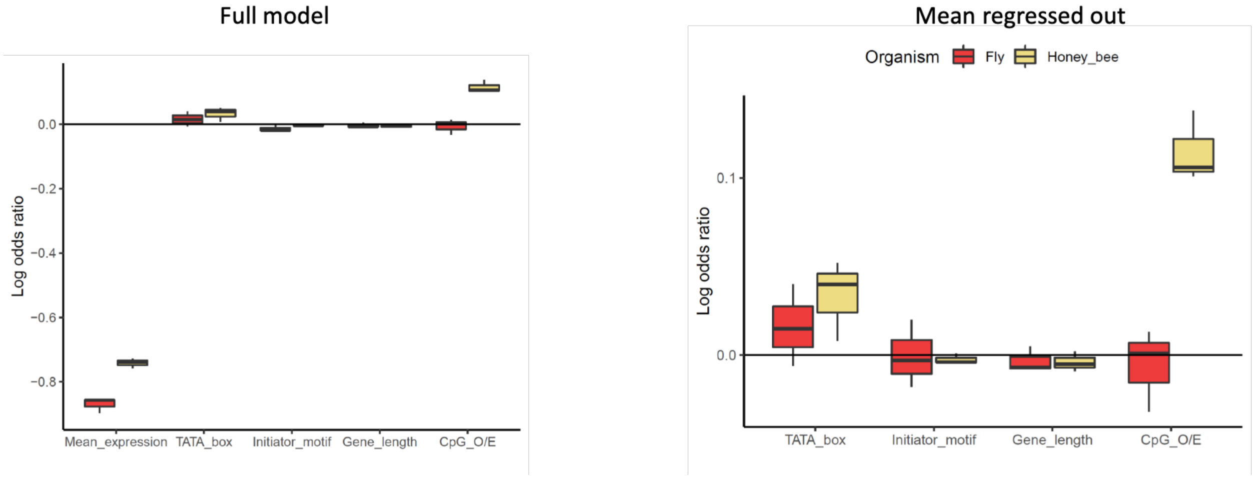

Supplementary Figure 2. Results of the analyses using only brain data sets. Data sets used include Shah et al. 2021, Miozzo and Nagoshi,unpublished, and Thackray et al. 2020 from *Apis mellifera*, and Liberti et al. 2019, Traniello et al. 2020 and Doublet et al. 2016 from *Drosophila melanogaster*.
